# Derivation and validation of a prediction score for postoperative delirium in geriatric patients undergoing hip fracture surgery or hip arthroplasty

**DOI:** 10.3389/fsurg.2022.919886

**Published:** 2022-08-19

**Authors:** Jiawei Shen, Youzhong An, Baoguo Jiang, Peixun Zhang

**Affiliations:** ^1^ Department of Orthopaedics and Trauma, Peking University People's Hospital, Beijing, China; ^2^ Department of Critical Care Medicine, Peking University People's Hospital, Beijing, China; ^3^ Key Laboratory of Trauma and Neural Regeneration, Peking University, Beijing, China; ^4^ National Center for Trauma Medicine, Beijing, China

**Keywords:** arthroplasty, complications, hip fracture, delirium, prognosis

## Abstract

**Introduction:**

Postoperative delirium is a common complication of patients undergoing hip fracture surgery or arthroplasty and is related to decreased survival time and physical function. In this study, we aim to build and validate a prediction score of postoperative delirium in geriatric patients undergoing hip fracture surgery or hip arthroplasty.

**Methods:**

A retrospective cohort of geriatric patients undergoing hip fracture surgery or hip arthroplasty was established. Variables of included patients were collected as candidate predictors of postoperative delirium. The least absolute shrinkage and selection operator (LASSO) regression and logistic regression were used to derive a predictive score for postoperative delirium. The accuracy of the score was evaluated by the area under the curve (AUC) of the receiver operating curve (ROC). We used bootstrapping resamples for model calibration. The prediction score was validated in an extra cohort.

**Results:**

There were 1,312 patients in the derivation cohort, and the incidence of postoperative delirium was 14.33%. Of 40 variables, 9 were identified as predictors, including preoperative delirium, cerebrovascular accident (CVA) with the modified Rankin scale, diabetes with a random glucose level, Charlson comorbidity index (CCI), age, application of benzodiazepines in surgery, surgical delay ≥2 days, creatine ≥90 μmol/L, and active smoker. The prediction score achieved a mean AUC of 0.848 in the derivation cohort. In the validation cohort, the mean AUC was 0.833. The prediction model was well-calibrated in the two cohorts.

**Conclusion:**

Based on retrospective data, a prediction score for postoperative delirium in geriatric patients undergoing hip fracture surgery or hip arthroplasty was derived and validated. The performance of the scoring system outperformed the models from previous studies. Although the generalization ability of the score needs to be tested in similar populations, the scoring system will enable delirium risk stratification for hip fracture patients and facilitate the development of strategies for delirium prevention.

## Introduction

Hip fracture is a major cause of mortality, morbidity, and economic burden in geriatric patients (1). As the population ages, the total incidence of hip fracture is increasing globally despite a decline in age-adjusted incidence (2); it is predicted that the incidence will increase to 6.26 by the year 2050 (3). Although the surgical technique has improved in recent years, a high incidence of 1-year mortality still exists (4). On the other hand, functional impairments including cognitive decline and reduced production of hormones (e.g., vitamin D, pro-inflammatory cytokines, and estrogens) often complicate the preoperative status of geriatric patients, leaving the patients more vulnerable to various postoperative complications.

Delirium, defined as “an acute confusional state,” is characterized by disturbance in attention and cognition (5). Postoperative delirium is a common complication with an incidence of 6.5%–55.9% in patients undergoing hip fracture surgery or hip arthroplasty (6). Delirium after surgery indicates a poor outcome with decreased survival time and impaired physical function (7). Moreover, postoperative delirium will increase hospital stay and postpone the functional recovery of patients (3). Preventing and controlling postoperative delirium are vital in clinical practice and have been set as a target for surgical quality improvement (8).

The early detection of postoperative delirium is important in delivering necessary care to patients at a higher risk. Although various prediction models have been developed for predicting postoperative delirium, previous studies were limited by a small sample size (9, 10) or a lack of patients who underwent hip arthroplasty (11, 12); in addition, there was no study with an appropriate sample size that had focused on the Chinese population (13) and most of the studies emphasized the evaluation of baseline mental or psychiatric presentations but omitted the disturbance of the microenvironment that may contribute to postoperative delirium (14, 15). As reported in previous studies, dysfunction of glucose metabolism and disturbance of microenvironments of neurons can contribute to the occurrence of delirium in geriatric patients (16, 17).

In the present study, by evaluating factors [electrolytes, albumin, blood urea nitrogen (BUN), etc.] that may affect the microenvironments of neurons and other possible predisposing factors of delirium, we aimed to derive and validate a prediction score for postoperative delirium in geriatric patients undergoing hip fracture surgery or arthroplasty.

## Materials and methods

This retrospective noninterventional study was approved by the ethics committee of Peking University People's Hospital and was performed in compliance with the Declaration of Helsinki.

### Study population

We retrospectively collected the electrical medical records of patients who were admitted to Peking University People's Hospital between January 2010 and December 2018. The inclusion criteria are as follows: (1) age ≥ 60 and (2) have received hip fracture surgery or hip arthroplasty. These patients were set as the derivation cohort of the prediction score. We further collected the medical records of patients who were admitted between January 2019 and March 2020 and included patients with the same criteria. These patients were set as the validation cohort.

### Outcome assessment and variable collection

The primary outcome of this study was the occurrence of delirium after surgery (during the recorded hospitalization). Delirium was identified using a clinically validated chart-based tool (18, 19) (see **Supplementary Table S1**) by screening the electronic medical records. In detail, two experienced physicians screened all medical records (including progress notes, nursing notes, surgical and anesthetic records, consulting notes, etc.) to search for evidence of the acute onset of confusional state (e.g., described as delirium, inattention, mental status change, disorientation, hallucinations, agitation, inappropriate behavior, etc.), patients with any of the listed evidence had a suggested diagnosis of delirium.

The following variables are collected as candidates for predictors of postoperative delirium: age, sex, body mass index (BMI), Carlson comorbidity index (CCI); previous history of major fractures (lower limb, pelvic, spinal), preoperative dementia, preoperative delirium, hearing loss, psychiatric disease, coronary heart disease, chronic heart failure, atrial fibrillation, hypertension, peripheral vascular disease, diabetes, chronic kidney disease, acute kidney injury, pre-urgery dialysis, hepatic failure, malignancy, alcohol abuse, active smoker, cerebral vascular accidents (CVA, evaluated with the modified Rankin Scale), chronic obstructive pulmonary disease (COPD), symptomatic pulmonary embolism (PE), preoperative pneumonia, American Anesthesia Society (ASA) score; type of fracture (femoral neck, intertrochanteric, subtrochanteric, periprosthetic, multiple hip fractures), type of surgery (intramedullary nail, cancellous screw, hemiarthroplasty, total arthroplasty, revision of arthroplasty), duration of surgical delay, duration of surgery, general anesthesia; high risk medications in surgery (benzodiazepines, opioids, nonsteroidal anti-inflammatory drugs), serum levels of glucose, sodium, BUN, creatine, albumin; and white blood cell count, hemoglobin level. All lab tests were performed on admission.

### Statistical analysis

Missing values were imputed when missing columns were less than 20%, and we used predictive mean matching for numeric variables, logistic regression for binary variables, and Bayesian polytomous regression for factor variables (>2 levels).

In the derivation cohort, candidate variables were selected using least absolute shrinkage and selection operator (LASSO) regression to minimize the overfitting and collinearity of variables. LASSO regression performs L1 regularization, in which a penalty value is equal to the magnitude of variable coefficients; larger penalties will force the smaller coefficients close to zero, thus resulting in a sparse model with fewer variables. These selected variables will enter logistic regression in the following analysis to build a prediction model. The significant predictors will be used to construct the prediction score for postoperative delirium with reference to a well-tested method (20); the accuracy of the prediction score was evaluated by the area under the curve (AUC) of the receiver operating curves (ROC). The accuracy of the prediction score was compared with the prediction models of two previous studies (12, 21).

We used 1,000 bootstrap resamples for model calibration (in both the derivation and validation cohort). Validation of the prediction score was performed by comparing the accuracy of the prediction in the derivation cohort and the validation cohort.

All statistical analyses were performed with R (version 4.0.2); R packages used were “MICE” (for processing missing values), “glmnet” (for LASSO regression), “glm” (for logistic regression), and “ggplot2” and “pROC” (for receiver operating characteristic curve depiction and calculation of the area under curves). *P* < 0.05 was considered statistically significant, and all statistical tests were two-sided.

## Results

### Characteristics of patients in the derivation cohort

**Figure 1:**
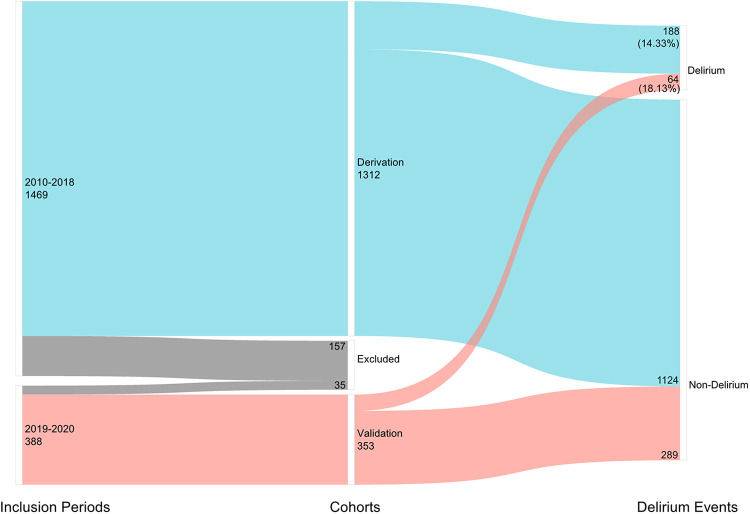
Number of included patients in different inclusion periods and the number of postoperative delirium cases.

A total of 535,037 records were screened in the electronic medical record system. Data of 1,312 patients were collected as the derivation cohort. Among these patients, 188(14.33%) had a suggested diagnosis of postoperative delirium (Figure 1). Baseline characteristics of patients are presented in Table 1. Most of the patients were in their 80s (46.1%) and women (896[68.29%]), with hypertension (793[60.44%]), diabetes (365[27.82%]), and coronary heart diseases (340[25.91%]) as the top three comorbidities.

**Table 1:** Baseline characteristics of patients in the derivation cohort who did or did not develop postoperative delirium.

Characteristic	Total	Delirium	
		No	Yes
	*n *= 1312	*N *= 1124	*n* = 188
		−85.67%	−14.33%
Age, years			
60–69	210(16.01)	204(18.15)	6(3.19)
70–79	375(28.58)	330(29.36)	40(21.28)
80–89	605(46.11)	502(44.66)	108(57.45)
≥90	122(9.30)	88(7.83)	34(18.09)
Sex			
Female (%)	896(68.29)	766(68.15)	130(69.15)
Male (%)	416(31.71)	358(31.85)	58(30.85)
BMI, kg/m^2^			
<20	430(32.77)	376 (33.45)	54(28.72)
20–25	591(45.05)	502(44.66)	89(47.34)
25–30	246(18.75)	207(18.42)	39(20.74)
>30	45(3.43)	39(3.47)	6(3.10)
CCI score	5(3–7)	5(3–6)	7(4–9)
History of major fractures	354(26.98)	299(26.60)	55(29.25)
Preoperative dementia	35(2.67)	7(0.53)	28(2.13)
Preoperative delirium	102(7.77)	49(4.36)	53(28.19)
Hearing loss	104(7.93)	90(8.01)	14(7.45)
Psychiatric disease	46(3.51)	39(3.47)	7(3.72)
Coronary heart disease	340(25.91)	293(26.07)	47(25.00)
Chronic heart failure	26(1.98)	20(1.78)	6(3.19)
Atrial fibrillation	106(8.08)	67(5.96)	39(20.74)
Hypertension	793(60.44)	694(61.74)	99(52.66)
Peripheral vascular disease	60(4.57)	55(4.89)	5(2.66)
Diabetes	365(27.82)	299(26.60)	66(35.11)
Chronic kidney disease	131(9.98)	97(8.63)	34(18.09)
Acute kidney injury	14(1.10)	8(0.71)	6(3.19)
Presurgery dialysis	12(0.92)	9(0.80)	3(1.60)
Hepatic failure	6(0.46)	5(0.44)	1(0.53)
Malignancy	206(15.71)	167(14.86)	39(20.74)
Alcohol abuse	33(2.52)	27(2.43)	6(3.17)
Active Smoker	93(7.09)	75(6.67)	18(9.57)
CVA with the Rankin scale			
4–5	15(1.14)	4(0.36)	11(5.85)
2–3	100(7.62)	41(3.65)	59(31.38)
0–1	166(12.65)	91(8.10)	75(39.89)
COPD	46(3.506)	43(3.825)	3(1.60)
Symptomatic PE	7(0.51)	5(0.44)	2(1.06)
Preoperative pneumonia	228(17.38)	198(17.62)	30(15.96)
ASA score	2(1–2)	2(1–2)	2(2–3)
Type of fracture			
Femoral neck	722(55.03)	661(58.81)	61(32.45)
Intertrochanteric	516(39.33)	396(35.23)	120(63.83)
Subtrochanteric	28(2.13)	21(1.87)	7(3.72)
Periprosthetic	20(1.53)	16(1.42)	4(2.13)
Multiple hip fractures	26(1.98)	19(1.67)	7(3.72)
Type of surgery			
Intramedullary nail	614(46.81)	562(50.00)	52(27.66)
Cancellous screw	73(5.56)	59(5.25)	14(7.45)
Hemiarthroplasty	470(35.82)	421(37.41)	49(26.06)
Total hip arthroplasty	148(11.28)	135(12.01)	13(6.91)
Revision of arthroplasty	7(0.53)	6(0.53)	1(0.52)
Surgical delay			
<24 h	85(6.48)	67(5.96)	8(4.26)
24–48 h	1055(80.4)	927(82.47)	138(73.40)
>48 h	172(13.1)	130 (11.57)	42(22.34)
Duration of surgery (h)	3.11 ± 0.91	3.11 ± 0.5	3.49 ± 0.88
General anesthesia	161(12.27)	139(12.37)	22(11.7)
High-risk medications in surgery			
Benzodiazepines	103(7.85)	35(3.11)	68(36.17)
Opioids	332(25.30)	289(25.71)	43(22.87)
NSAIDs	258(19.66)	222(19.75)	36(19.14)
Glucose level for patients with diabetes (mmol/L)	8.77 ± 1.64	7.96 ± 2.55	8.43 ± 1.38
Sodium (mmol/L)	136.09 ± 16.21	135.53 ± 17.38	139.47 ± 3.67
BUN (mg/dL)	6.69(5.16–8.8)	6.41(5.00–8.41)	7.81(6.29–10.88)
Creatine (mmol/L)	69(56–94)	62(59–107)	70(55–95)
Albumin (mmol/L)	35.62 ± 4.89	35.82 ± 5.02	34.4 ± 4.85
White blood cell count (×10^9^/L)	8.06 ± 2.73	8.00 ± 2.53	8.39 ± 3.66
Hemoglobin (g/L)	119(101–131)	110(92–130)	97(90–113)

Data are given as a number (%), mean + standard deviation, or median (interquartile range).

ADL, activities of daily living; BMI, body mass index; BUN, blood urea nitrogen; CCI, Carlson comorbidity index; CVA, cerebrovascular accident; COPD, chronic obstructive pulmonary disease; NSAIDs, nonsteroidal anti-inflammatory drugs; PE, pulmonary embolism.

A large majority of patients had femoral neck fracture (722[55.03%]), followed by intertrochanteric (516[39.33%]), subtrochanteric (28[2.13%]), multiple locations of the hip (26[1.98%]), and periprosthetic fractures (20[1.53%]). Most patients received intramedullary nail fixation (614[46.53%]), followed by hemiarthroplasty (470[35.82%]), total hip arthroplasty (148[11.28%]), cancellous screw (73[5.56%]), and revision of arthroplasty (7[0.53%]).

### Selection of variables as predictors

With LASSO regression, 12 out of 40 variables were selected (see **Supplementary Figure S1**). The selected variables include pre-operative delirium, CVA with the modified Rankin scale, age, CCI, random glucose levels of patients with diabetes, application of benzodiazepines in surgery, surgical delay, creatine level, active smoker, general anesthesia, serum BUN level, and albumin level.

All 12 variables were then analyzed with the logistic regression model. There were nine variables that remained in the model: preoperative delirium (OR 4.21, 95% CI 3.25–9.14, *P* < 0.001), CVA with the modified Rankin scale (4–5, OR 3.17, 95% CI 1.16–5.06; 2–3, OR 2.25, 95% CI 1.26–4.29, *P* < 0.001), diabetes with a random glucose level (>13 mmol/L, OR 2.43, 95% CI 1.32–2.99; 8–13, OR 1.36, 95% CI 1.15–1.67, *P* = 0.023), CCI (≥9, OR 2.43, 95% CI 1.32–2.99; 6–8, OR 1.29, 95% CI 1.03–2.52, *P* = 0.008), age (≥80, OR 1.87, 95% CI 1.25–2.50; 70–79, OR 1.11, 95% CI 1.07–2.19, *P* = 0.021), application of benzodiazepines in surgery (OR 1.44, 95% CI 1.24–3.17, *P* = 0.036), surgical delay ≥2 days (OR 1.15, 95% CI 1.13–1.28, *P* = 0.012), creatine ≥90 μmol/L (OR 1.09, 95% CI 1.02–1.13, *P* = 0.034), and active smoker (OR 1.05, 95% CI 1.04–1.94, *P* = 0.042) (Table 2).

**Table 2:** Multivariate regression of postoperative delirium.

Variable	OR	95% CI	*P*
Preoperative delirium	4.21	3.25–9.14	<0.001
CVA with the modified Rankin scale			
4–5	3.17	1.16–5.06	<0.001
2–3	2.25	1.26–4.29	
0–1^a^	–		
Diabetes with random glucose level			0.023
>13 mmol/L	2.43	1.32–2.99	
8–13 mmol/L	1.36	1.15–1.67	
<8 mmol/L^b^	–		
CCI score			0.008
≥9	2.32	1.69–4.83	
6–8	1.29	1.03–2.52	
≤5	–		
Age (years)			
≥80	1.87	1.25–2.50	0.021
70–79	1.11	1.07–2.19	
60–69	–		
Application of Benzodiazepines in surgery	1.44	1.24–3.17	0.036
Surgical delay ≥2 days	1.15	1.13–1.28	0.012
Creatine ≥90 μmol/L	1.09	1.02–1.13	0.034
Active smoker	1.05	1.04–1.94	0.042
General anesthesia	1.32	0.32–1.63	0.751
BUN >8.5 mmol/L	1.15	0.62–1.77	0.812
Albumin ≤30 g/L	1.08	0.86–1.26	0.623

^a^ Without CVA.

^b^ Without diabetes.

ADL, activities of daily living; BUN, blood urea nitrogen; CCI, Carlson comorbidity index; CI, confidence interval; CVA, cerebrovascular accident; OR, odds ratio.

### Derivation of the prediction score

**Figure 2:**
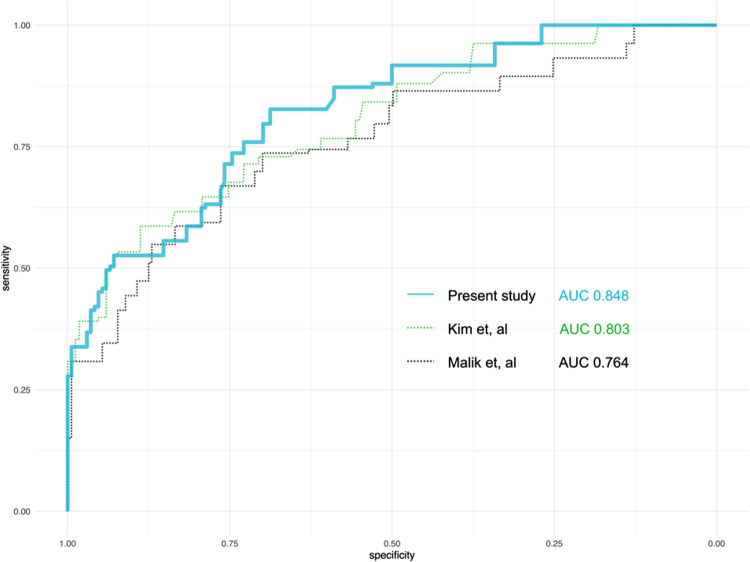
Comparison of the performance of the predicting models from the present study and two previous studies. AUC, area under the curve.

Using the nine variables selected in the last section, a prediction score was derived (Table 3). With bootstrapping, the mean AUC of the prediction score in the derivation cohort was 0.848 (95% CI 0.72–0.90), higher than the models of previous studies (Figure 2). The range of the score and the estimated probability of postoperative delirium were recorded in **Supplementary Table S2**.

**Table 3:** Prediction score of postoperative delirium for patients after hip fracture surgery or hip arthroplasty.

Variable	Score
Preoperative delirium	4
CVA with the modified Rankin scale	
≥4	3
2–3	2
0–1^a^	0
Diabetes with random glucose level	
>13 mmol/L	2
8–13 mmol/L	1
<8 mmol/L^b^	0
CCI score	
≥9	2
6–8	1
≤5	0
Age (years)	2
≥80	1
70–79	0
60–69	
Application of benzodiazepines in surgery	1
Surgical delay ≥2 days	1
Creatine ≥90 μmol/L	1
Active smoker	1

^a^Without CVA.

^b^Without diabetes.

ADL, activities of daily living; CCI, Carlson comorbidity index; CVA, cerebrovascular accident.

### Validation of the prediction score

**Figure 3:**
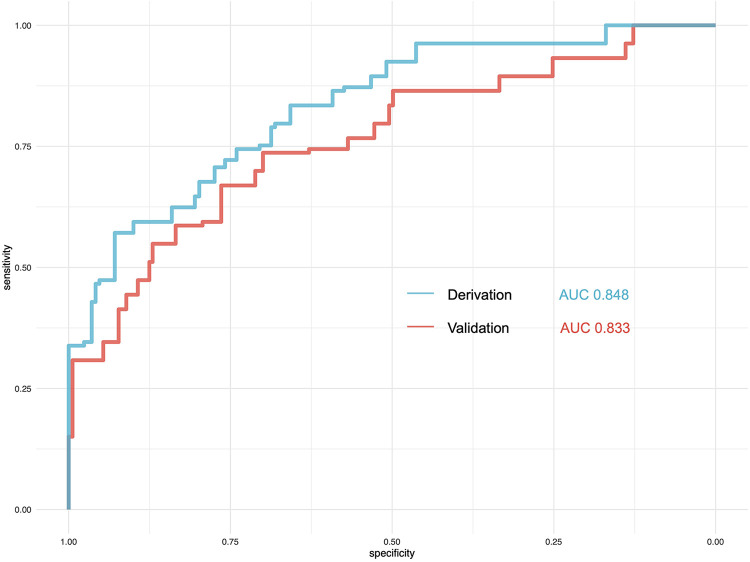
Comparison of the performance of the prediction model in the derivation and validation cohorts. AUC, area under the curve.

**Figure 4:**
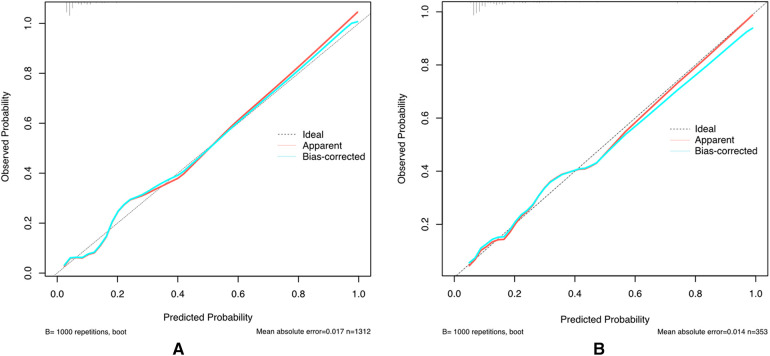
Calibration plot for the prediction model in the derivation and validation cohorts.

We then use the validation cohort [with 353 patients and 64 (18.31%) with postoperative delirium] (Table 4) to test the performance of the prediction score model. Through 1,000 times bootstrapping, the mean AUC of the model was 0.833 (95% CI 0.68–0.89) (Figure 3), and the model was well-calibrated in both the derivation and validation cohorts (Figure 4). The prediction score had similar accuracy to postoperative delirium in the derivation and validation cohorts.

**Table 4:** Baseline characteristics of patients in the validation cohort who did or did not develop postoperative delirium.

Characteristic	Total	Delirium	
	*n *= 353	No	Yes
		*N *= 289	*n *= 64
		−81.87%	−18.13%
Age, years			
60–69	51(14.45)	49(16.96)	2(3.13)
70–79	78(22.10)	66(22.84)	10(15.63)
80–89	165(46.74)	135(46.71)	30(46.88)
≥90	59(16.71)	39(13.49)	22(34.38)
Sex			
Female (%)	235(66.57)	196(67.82)	39(60.94)
Male (%)	118(33.43)	93(32.18)	25(39.06)
BMI, kg/m^2^			
<20	116(32.86)	96(33.21)	20(31.25)
20–25	160(45.33)	131(45.33)	29(45.31)
25–30	64(18.13)	51(17.65)	13(20.31)
>30	13(3.68)	11(3.81)	2(3.13)
CCI score	6(3–6)	5(3–6)	5(3–9)
History of major fractures	89(25.21)	71(24.57)	18(28.13)
Preoperative dementia	29(8.22)	23(7.96)	6(9.38)
Preoperative delirium	16(4.53)	9(3.11)	7(10.94)
Hearing loss	21(5.95)	19(5.38)	2(3.13)
Psychiatric disease	13(3.68)	11(3.81)	2(3.13)
Coronary heart disease	82(23.23)	67(23.18)	15(23.44)
Chronic heart failure	6(1.70)	5(1.73)	1(1.56)
Atrial fibrillation	27(7.65)	18(6.23)	9(14.06)
Hypertension	207(58.64)	173(59.86)	34(53.13)
Peripheral vascular disease	16(4.53)	16(5.54)	0(0)
Diabetes	99(28.05)	70(24.22)	29(45.31)
Chronic kidney disease	34(9.63)	20(6.92)	14(21.88)
Acute kidney injury	8(2.27)	5(1.73)	3(4.69)
Presurgery dialysis	6(1.70)	4(1.38)	2(3.13)
Hepatic failure	5(1.42)	3(1.04)	2(3.13)
Malignancy	51(14.45)	39(13.49)	12(18.75)
Alcohol abuse	9(2.55)	7(2.42)	2(3.13)
Active smoker	26(7.37)	22(7.61)	4(6.25)
CVA with the Rankin scale			
4–5	9(2.55)	5(1.73)	4(6.25)
2–3	23(6.52)	7(2.42)	16(25.00)
0–1	11(3.12)	5(1.73)	6(9.38)
COPD	13(3.68)	6(2.08)	7(10.94)
Symptomatic PE	1(0.28)	1(3.45)	0(0)
ASA score	2(1–2)	2(1–2)	2(1–3)
Preoperative pneumonia	57(16.15)	34(11.76)	70(37.23)
Type of fracture			
Femoral neck	190(53.82)	172(59.52)	18(28.13)
Intertrochanteric	145(41.08)	101(34.95)	44(68.75)
Subtrochanteric	6(1.70)	4(1.38)	2(3.13)
Periprosthetic	5(1.42)	5(1.73)	0(0)
Multiple hip fractures	7(1.98)	7(2.42)	0(0)
Type of surgery			
Intramedullary nail	140(39.65)	102(35.29)	38(59.38)
Cancellous screw	45(12.75)	36(12.46)	9(14.06)
Hemiarthroplasty	121(34.28)	116(40.14)	5(7.81)
Total hip arthroplasty	42(11.90)	32(11.07)	10(15.63)
Revision of arthroplasty	5(1.42)	3(1.04)	2(3.13)
Surgical delay			
<24 h	22(6.23)	22(7.61)	0(0)
24–72 h	285(80.74)	34(11.76)	52(81.25)
>72 h	46(13.03)	233 (80.62)	12(18.75)
Duration of surgery	3.15 ± 0.93	3.08 ± 0.85	3.48 ± 0.81
General anesthesia	35(9.92)	27(9.34)	8(12.5)
High-risk medications in surgery			
Benzodiazepines	59(16.71)	26(8.99)	33(51.56)
Opioids	233(66.01) 78(22.10)	167(57.79) 63(21.80)	12(18.75)
NSAIDs			15(23.44)
Glucose level for patients with diabetes (mmol/L)	8.69 ± 1.57	7.14 ± 3.44	8.85 ± 1.50
Sodium	134.02 ± 17.28	135.25 ± 18.72	136.39 ± 9.63
BUN	7.84(5.25–8.73)	6.38(5.02–8.08)	7.93(6.45–10.25)
Creatine	58(54–89)	67(55–85)	69(59.05–99)
Albumin	35.66 ± 4.42	35.95 ± 5.65	35.26 ± 4.92
White blood cell count	8.55 ± 2.97	7.98 ± 2.56	8.26 ± 3.77
Hemoglobin	119(99–127)	122(104–133)	105(88–104.8)

Data are given as a number (%), mean + standard deviation, or median (interquartile range).

ADL, activities of daily living; BMI, body mass index; BUN, blood urea nitrogen; CCI, Carlson comorbidity index; CVA, cerebrovascular accident; COPD, chronic obstructive pulmonary disease; NSAIDs, nonsteroidal anti-inflammatory drugs.

## Discussion

Based on the retrospective data, a prediction score for postoperative delirium in geriatric patients undergoing hip fracture surgery or hip arthroplasty was derived and validated. In the derivation and validation cohorts, the accuracy of the score was satisfactory. The score consists of nine prediction factors (preoperative delirium, CVA with the modified Rankin scale, random glucose levels of patients with diabetes, CCI score, age, application of benzodiazepines in surgery, surgical delay, creatine level, active smoker) that are easily available in clinical practice. Moreover, the scoring system also included functional evaluation of CVA patients and disturbances of microenvironments (random glucose levels of diabetes, creatine levels), which were not reported in previous studies. In the derivation cohort, the suggested incidence of delirium was 14.33%, which was close to publications (9, 22) with similar patient composition; some of the predictors were consistent with previous studies (6, 12).

### Predisposing factors that related to delirium

Preoperative delirium was identified as a significant predictor of postoperative delirium. As reported by a previous study, postoperative delirium had a high incidence in patients diagnosed with preoperative delirium (23) (up to 60%). However, in most of the related studies, preoperative delirium has been excluded in the process of patient selection (9, 24). This may be explained as an attempt to rule out prevalent delirium cases from new-onset postoperative delirium cases. However, excluding this group of patients will underestimate the incidence of postoperative delirium; on the other hand, preoperative delirium may not persist after surgery. In the derivation cohort, 102(7.77%) patients had preoperative delirium, and 53(51.96%) of these patients were in a delirium state postoperatively, while 48.04% of these patients had no delirium after the surgery. It is necessary to include patients with preoperative delirium in the prediction model to evaluate the risk of the postoperative state of delirium.

Although delirium may affect patients of various ages, geriatric patients suffer much more frequently and more severely from it. In an observational study that focused on preoperative and postoperative delirium, hip fracture patients older than 80 years were more likely to be complicated with delirium (25). The cause of the high incidence of delirium in geriatric patients may be attributed to accumulated neuron injury accompanied by aging. At the same time, cerebrovascular accidents (CVA) can render geriatric patients more susceptible to delirium in stress states like trauma or surgery (26); moreover, impaired daily life function renders patients with a higher risk of delirium after stroke (27). In the present study, CVA with higher degrees of disability (evaluated with the modified Rankin scale) was also identified as a predictor of postoperative delirium, which indicates that the severity of neuroinjury brought by the CVA was associated with the risk of delirium.

Diabetes has been proved to be a predisposing factor for dementia (including vascular disease or Alzheimer's disease-related dementia) (28). Similarly, we observed an association between uncontrolled glucose levels and delirium episodes. It is rational to hypothesize that long-lasting vascular damage and inflammatory response in diabetes (29) will affect neuro functions.As supported by pathophysiological studies, elevated intracellular glucose levels will inhibit the mitochondrial function of neurons (30). The role of deregulated glucose metabolism in the cerebral spinal fluid has been reported to be related to cognitive dysfunction in mice and patients with delirium (31). Similarly, it has been reported that high variability of the serum glucose level was related to delirium in patients after aortic dissection surgery (15), which suggested a possible role of glucose management in delirium prevention for hip fracture patients.

High CCI has been proved (32) to be related to higher mortality and complications after hip fracture surgery. The CCI evaluated the number of comorbidities and the severity of the concomitant diseases, which was consistent with the result of Zhao et al. that the frailty of patients was a predictor of delirium after surgery (33). Another identified predictor in our study was active smoking. Physiological research has discovered that nicotine, the main component of tobacco, can affect the cerebral microvascular function and can prompt the process of atherosclerosis (34), which may further lead to delirium. Similarly, in our cohort, active smokers were liable to new-onset delirium, which may be attributed to the acetylcholine deficiency (35) caused by acute abstinence before surgery. In addition, disturbances of the internal environment caused by comorbidities or stress from the injury, presented with factors like high creatine levels, may also be interfering with the normal functioning of neurons and cause delirium (21, 33).

### Medical interventions related to delirium

Benzodiazepines have been identified as a risk factor for delirium (36). When compared with other sedatives like propofol or dexmedetomidine, benzodiazepines are more efficient in affecting neurotransmitter concentrations and impairing the quality of sleep *via* slow-wave sleep suppression, which may finally lead to delirium (37). The fact that patients treated with benzodiazepines were more liable to delirium made the choice of sedatives even more critical in the surgery. On the contrary, opioids, which were reported as a risk factor for delirium in other studies (38, 39), were not included in the scoring system, which implies that the effect of sedatives can vary between different types of patients.

There is plenty of evidence that surgical delay is related to poor functional outcomes and mortality (40). The multivariable logistic regression in our study shows that surgical delay of >2 days was an independent risk factor for postoperative delirium, which indicates that, regardless of baseline conditions of patients, the delaying of the timely surgical intervention itself can affect cognitive function. A similar result was reported by Pioli et al. hat delay of surgery for hip fractures will increase the risk of delirium in patients with a history of cognitive impairments (41).

In view of the prevalence and prognosis of delirium after hip fracture surgery and arthroplasty, a validated scoring system will facilitate risk stratification for patients with identifiable risk factors of delirium at the time of hospital admission. In addition, timely intervention of risk factors will prevent about 30% of delirium episodes (42), making the search for risk factors and the development of prediction scores more reasonable. Based on the predictive variables identified in the present cohort, future studies can further explore the pathophysiological mechanisms of postoperative delirium in patients with hip fractures. What is more, interventional strategies can also be developed accordingly.

Currently, the treatment of delirium is mainly multidisciplinary, which focuses on interventions for various risk factors of delirium (43). Even though the multidisciplinary approach has not shown an overall significant improvement in mortality or hospitalization in random contrast trials, one of the studies suggested that this approach does reduce the time of recovery for cognitive functions (44). Given the lack of interventions that are supported by high-quality evidence, a clinician should direct more resources toward those with a higher risk of postoperative delirium and at least address correctable physiological variables in a timely manner.

## Limitations

The present study is limited by several issues. First, as a result of the retrospective nature of this study, we used chart-based tools instead of a validated screening tool for delirium [e.g., Confusion Assessment Method (45)]. However, as evaluated by a previous study (18), this tool and Confusion Assessment Method had an overall good inter-rater agreement (with an agreement of 82%, kappa = 0.41). Second, for benzodiazepines, we did not compare the difference between continuous infusion or boluses to the occurrence of postoperative delirium because only continuous infusion was related to delirium in one study (46). Finally, the score was derived from a single-center cohort; characteristics of the derivation cohort may not fit the whole population. The prediction score still needs to be validated in cohorts with more sufficient sample sizes in the future.

## Conclusions

Based on retrospective data, a prediction score for postoperative delirium in geriatric patients undergoing hip fracture surgery or hip arthroplasty was derived and validated. The performance of the scoring system outperformed the models from previous studies. Although the generalization ability of the score needs to be tested in similar populations, the scoring system will enable delirium risk stratification for hip fracture patients and facilitate the development of strategies for delirium prevention.

## Data Availability

The raw data supporting the conclusions of this article will be made available by the authors without undue reservation.
