# Supplementary material for: Derivation and validation of a prediction score for postoperative delirium in geriatric patients undergoing hip fracture surgery or hip arthroplasty
Source: Front Surg. 2022 Aug 19;9:919886. doi: 10.3389/fsurg.2022.919886 (PMC9437918; doi:10.3389/fsurg.2022.919886)
Supplement: Supplementary file 1 [file Table_1_v1.doc]

Table S1. Chart-Based Instrument for Delirium During Hospitalization

1. Is there any evidence from the chart of acute confusional state (e.g., delirium, mental status change, inattention, disorientation, hallucinations, agitation, inappropriate behavior, etc.)? Review the entire medical record, including progress notes, nursing notes, consult notes, etc.

****Yes GO TO NEXT SECTION

****No

****Uncertain

2. (If yes) What is the source of information about the ﬁrst episode of acute confusion?

****Nurse’s notes

****Physician’s progress notes

****Other (specify): __________________

****NA

3. (If yes) Approximate time of onset of ﬁrst episode of acute confusion? Check nurse’s notes, progress notes, orders, laboratories, for earliest time recorded referable to the event.

Date: ___ ___ / ___ ___ / ___ ___ month day year ****NA

Time: ___ ___ : ___ ___ ****Uncertain ****NA

4. Describe each reference to acute confusion in the chart, verbatim:

| Date | Time | Source | Description(verbatim, in detail) |
| --- | --- | --- | --- |
|  |  |  |  |
|  |  |  |  |
|  |  |  |  |
|  |  |  |  |

5. Review all notes. Was acute confusion noted by:

a. Any physician?

Specify all: _______________________________ _______________________________

****Yes ****No ****Uncertain ****NA 9

b. Any nurse?

****Yes ****No ****Uncertain ****NA

6. What was the total duration (in days) of acute confusion (as determined by all references to confusion in chart):

Days ___ ___ ___ ****Uncertain ****NA

7.

a. Was there any evidence of reversibility or improvement of acute confusion during the hospitalization?

****Yes ****No ****Uncertain ****NA

b. (If yes) Please describe evidence:

*NA*: not applicable.

*Modified from Inouye, S. K., et al. (2005). "A chart-based method for identification of delirium: validation compared with interviewer ratings using the confusion assessment method." J Am Geriatr Soc* ***53****(2): 312-318.*

Table S2. Prediction score and the estimated probability of post-operative delirium

| Total prediction score | Estimated probability of post-operative delirium |
| --- | --- |
| 0 | 5.2% |
| 1 | 6.3% |
| 2 | 10.8% |
| 3 | 26.7% |
| 4 | 33.3% |
| 5 | 45.2% |
| 6 | 61.1% |
| 7 | 62.4% |
| 8 | 65.1% |
| 9 | 67.4% |
| 10 | 69.5% |
| 11 | 70.3% |
| 12 | 72.8% |
| 13 | 74.5% |
| 14 | 76.1% |
| 15 | 77.1% |
| 16 | 80.7% |
| 17 | 82.2% |

Table S3. Confusion matrix of prediction score in derivation and validation cohorts.

| Cohorts | Accuracy | Sensitivity | Specificity | True Negative | False Negative | True  Positive | False  Positive |
| --- | --- | --- | --- | --- | --- | --- | --- |
| Derivation | 85.5% | 84.9% | 85.1% | 86.7% | 13.3% | 83.2% | 16.8% |
| Validation | 82.1% | 81.1% | 83.9% | 81.3% | 19.7% | 84.4% | 15.6% |

Fig. S1.

Title Variable selection by least absolute shrinkage and selection operator (LASSO) regression model

1. Coefficient profile of candidate variables.
2. Lambda selection with 10-fold cross-validation (lambda value of mimimun+1 standard deviation was selected).
